# EVIDENCE-BASED REVIEWS & RECOMMENDATIONS FROM THE BRAZILIAN SOCIETY OF SHOULDER AND ELBOW SURGERY: EARLY VERSUS LATE REPAIR FOR TRAUMATIC ROTATOR CUFF TEARS

**DOI:** 10.1590/1413-785220263401e308952

**Published:** 2026-08-21

**Authors:** Paulo Henrique Schmidt Lara, Paulo Santoro Belangero, Márcio Schiefer, Leonardo Zanesco, Marcos Rassi Fernandes, Joel Murachovsky, João Felipe de Medeiros, Eduardo Angeli Malavolta

**Affiliations:** 1 Escola Paulista de Medicina, UNIFESP, Sao Paulo, SP, Brazil.; 2 Instituto Nacional de Traumatologia e Ortopedia Jamil Haddad (INTO), Rio de Janeiro, RJ, Brazil.; 3 Universidade de Sao Paulo, Faculdade de Medicina, Hospital das Clinicas HCFMUSP, Sao Paulo, SP, Brazil.; 4 Universidade Federal de Goias, Faculdade de Medicina, Goiania, GO, Brazil.; 5 Faculdade de Medicina do ABC, Santo Andre, SP, Brazil.; 6 Universidade Federal do Rio Grande do Norte, Natal, RN, Brazil.

**Keywords:** Rotator Cuff Tear, Trauma, Operative Time, Tendon Injuries, Shoulder, Ruptura do Manguito Rotador, Trauma, Duração da Cirurgia, Traumatismos dos Tendões, Ombro

## Abstract

**Introduction:**

Traumatic rotator cuff tears are acute structural injuries to one or more rotator cuff tendons caused by an identifiable trauma, generally associated with the abrupt onset of pain, weakness, and shoulder dysfunction. They must be distinguished from chronic degenerative tears and from acute injuries superimposed on previously degenerated tendons. The optimal timing of surgical repair remains debated.

**Methods:**

A systematic review was conducted in PubMed, Embase, Cochrane Library, and the Virtual Health Library (VHL) up to March 2026 to evaluate whether early repair is more effective than late repair for the treatment of traumatic rotator cuff tears, with respect to clinical and functional outcomes, retear, and return to pre-injury activities. The certainty of evidence was assessed with the GRADE approach.

**Results:**

Three cohort studies were included. The definition of "early" repair is not standardized in the literature – some authors consider it up to 3 weeks and others up to 6 months – so studies defining "early" as up to 3–4 months were included, which represents a potential source of bias. Functional outcomes were similar between early and late repair, whereas early repair was associated with and a lower likelihood of requiring tendon grafting in larger tears. The certainty of evidence was very low.

**Conclusion:**

The surgeon should individually assess patient- and disease-related characteristics to make the best treatment decision. Given the risk of irreparability and longer rehabilitation, the project recommends that, after a traumatic tear, repair be performed as early as possible. ***Level of evidence II; Systematic review.***

## RESUMO

**Introdução:**

A ruptura traumática do manguito rotador consiste na lesão estrutural aguda de um ou mais tendões do manguito, ocasionada por trauma identificável, em geral associada ao início abrupto de dor, fraqueza e disfunção do ombro, devendo ser distinguida das rupturas degenerativas crônicas e das lesões agudas sobre tendões previamente degenerados. O momento ideal do reparo cirúrgico permanece controverso.

**Métodos:**

Realizou-se revisão sistemática da literatura nas bases de dados PubMed, Embase, Cochrane Library e Biblioteca Virtual em Saúde (BVS) até março de 2026, para avaliar se o reparo precoce é mais efetivo do que o reparo tardio no tratamento das lesões traumáticas do manguito rotador, em relação aos desfechos clínico-funcionais, rerruptura e retorno às atividades pré-lesão. A certeza da evidência foi avaliada pela abordagem GRADE.

**Resultados:**

Foram incluídos três estudos de coorte. O termo "precoce" não está padronizado na literatura – alguns autores consideram até 3 semanas e outros até 6 meses – de modo que foram incluídos estudos que definiram "precoce" como até 3 a 4 meses, o que representa potencial fonte de viés. Os desfechos funcionais foram semelhantes entre reparo precoce e tardio, enquanto o reparo precoce associou-se a recuperação mais rápida e menor necessidade de enxertia tendínea nas lesões maiores. A certeza da evidência foi muito baixa.

**Conclusão:**

O cirurgião deve avaliar, de maneira individualizada, as características do paciente e da enfermidade para a tomada da melhor decisão de tratamento. Devido ao risco de irreparabilidade e ao maior tempo de reabilitação, este projeto recomenda que, após uma ruptura traumática, o reparo seja realizado o mais brevemente possível. ***Nível de evidência II; Revisão sistemática.***

## INTRODUCTION

Traumatic rotator cuff tear is an acute structural injury to one or more rotator cuff tendons, caused by an identifiable trauma and generally associated with the abrupt onset of pain, weakness, and shoulder dysfunction. It should be distinguished from chronic degenerative tears and from acute injuries superimposed on previously degenerated tendons.^1–3^

Treatment may be conservative or surgical. Surgical treatment is generally indicated for complete tears, for symptomatic and functionally disabling lesions, and for potentially repairable tears.^4–5^

Currently, the most commonly used surgical treatment is arthroscopic repair with suture anchors; however, the repair may also be performed through open or combined (open and arthroscopic) approaches.^6–7^ There is considerable controversy regarding the "ideal timing" for surgery. Many surgeons argue that early surgery (before 3 months after injury) offers advantages such as less tendon retraction, a better chance of anatomical reduction, less progression of fatty degeneration, and lower surgical complexity. Surgeons who favor delayed surgery (3 months or more after injury) argue that this interval allows assessment of whether surgery is truly necessary and whether conservative treatment has succeeded, and they report that the functional outcome of late surgery is comparable to that of early surgery.^6,7^

Accordingly, this consensus was developed to evaluate whether early repair is more effective than late repair for the treatment of traumatic rotator cuff tears, with respect to clinical-functional outcomes, re-tear, and return to pre-injury activities.

This study was conducted at the initiative of the Brazilian Society of Shoulder and Elbow Surgery with the participation of Cochrane Brazil.

### Research Question

Is early repair more effective than late repair for the treatment of traumatic rotator cuff tears?

## METHODS

- Databases searched: PubMed, Embase, Cochrane Library, and the Virtual Health Library (VHL).
- Descriptors: As described in Appendix 1.
- Search period: Until March 2026.
- Study selection: Updated systematic reviews (SRs) with adequate methodological quality were prioritized. In the absence of suitable SRs, randomized controlled trials (RCTs) and observational studies were considered.

## RESULTS

**Figure 1:**
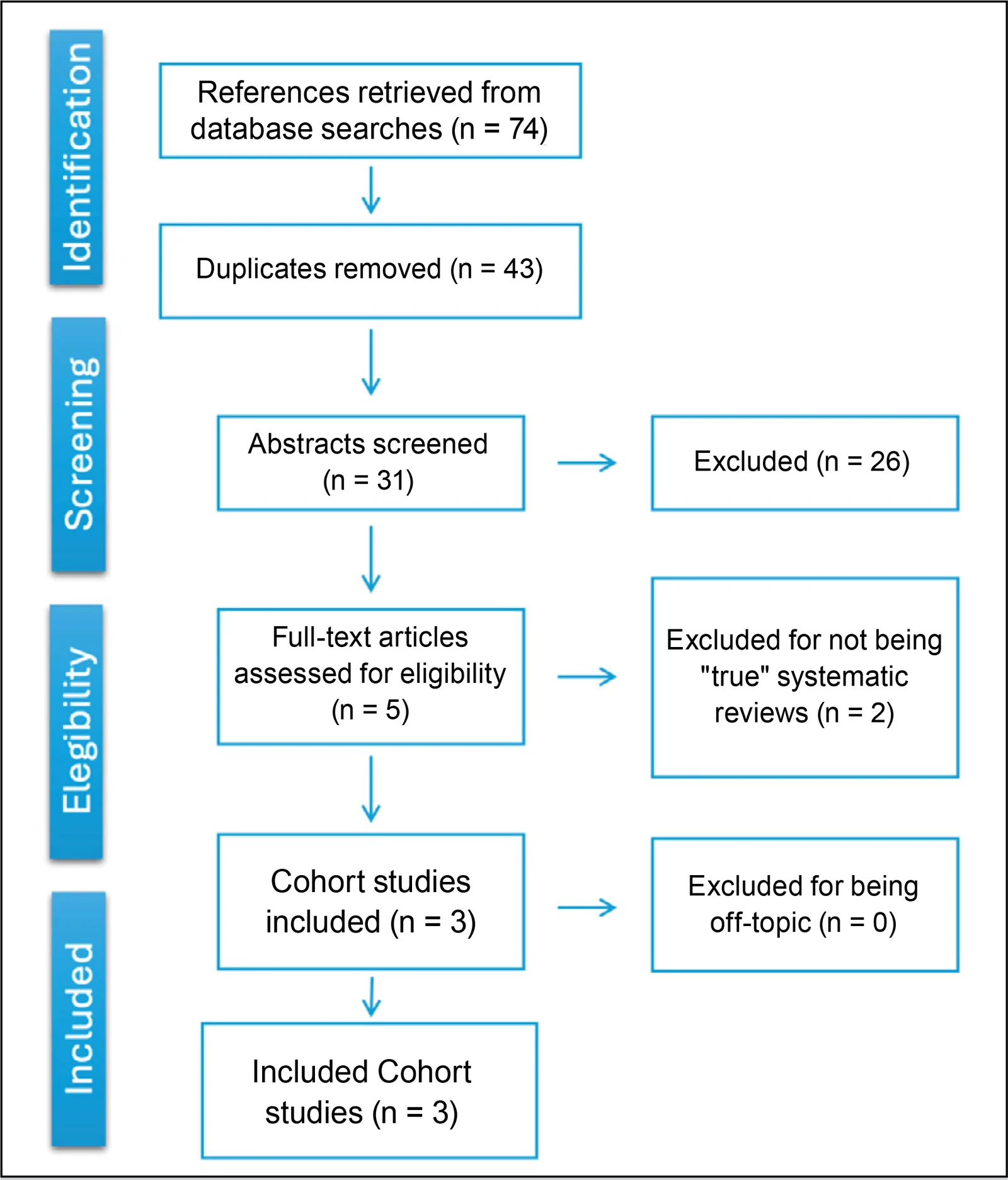
Flow diagram of the literature search.

The search retrieved 74 references (27 in PubMed, 17 in Embase, 30 in the Cochrane Library, and 0 in the Regional Portal BVS). After the initial assessment, 43 duplicate records were removed. Following abstract screening, 26 studies were excluded, leaving five studies for full-text evaluation. Two systematic reviews on the topic were assessed but not included (Figure 1).

Pinto et al.,^8^ in a systematic review, included 20 studies (cohort studies, case reports, case series, and prospective and retrospective studies) and reported that the literature is heterogeneous and that the existing evidence is low, being based on low-quality clinical and observational studies. This review was not included in the present consensus because the authors pooled several different study types. Baur et al.,^9^ in a very low-quality systematic review, included 13 studies and 871 participants (retrospective and prospective studies, cohorts, case series, and case reports) and performed a meta-analysis combining markedly heterogeneous designs. The authors concluded that early repair is associated with a lower re-tear rate and better functional outcomes. This review was also not included, for the same reason.

### Included studies

Three cohort studies were ultimately included. It should be noted that the term "early" is not fully defined in the literature: some authors consider it up to 3 weeks and others up to 6 months.^7,10,11^ To avoid omitting the scarce available evidence, studies defining "early" as up to 3 to 4 months were included, which is acknowledged as a source of bias in this consensus.

Patel et al.,^7^ in a retrospective cohort study, evaluated 40 patients to determine whether the time to surgery influenced outcomes in symptomatic traumatic rotator cuff tears. Patients were divided into an early-repair group (surgery within 4 months of injury) and a late-repair group (surgery after 4 months). The outcomes analyzed were functional scores (Oxford Shoulder Score, Constant Score, and EQ-5D), time to full recovery, re-tear rate, and the need for interpositional grafting when the tendon could not be directly repaired. There was no significant difference in functional outcomes between groups; however, the late-repair group had a longer recovery time (early: approximately 14 months; late: approximately 33.8 months). For tears ≥ 3 cm, interpositional grafting was required in 44% of cases in the late group and in none in the early group. Only one symptomatic re-tear occurred in the series, in the early group.

The authors concluded that early repair was associated with faster recovery and a lower need for grafting in larger tears, although no difference in final functional scores between early and late repair was observed at intermediate follow-up.^7^

Zhaeentan et al.,^10^ in a prospective cohort study, evaluated 75 shoulders from 58 male patients operated on for traumatic rotator cuff tears between 1999 and 2011. The authors compared open repair performed early (< 3 months after injury; 39 shoulders) versus late (> 3 months after injury; 36 shoulders). The mean follow-up was 56 months, and the mean time from injury to repair was 16 weeks (range 3 to 104 weeks). The outcomes evaluated were the Western Ontario Rotator Cuff Index (WORC), the Oxford Shoulder Score, the Constant Score, the EQ-5D, patient satisfaction, repair integrity on MRI, re-tear, muscle atrophy, fatty infiltration, and osteoarthritis. No significant difference was found between groups in any of the main scores. The re-tear rate was 24%, with 9 cases in each group. The authors concluded that surgical treatment of symptomatic traumatic rotator cuff tears, even when performed more than 3 months after injury, can provide good functional results and high satisfaction, provided that the tendon remains technically repairable.^10^

Petersen et al.^11^, in a prospective cohort study, evaluated 36 patients with traumatic rotator cuff tears, with a minimum follow-up of 9 months (mean 31 months; range 9 to 71 months). The outcomes assessed were the UCLA End-Result and ASES functional scores, the time between injury and repair, lesion size, preoperative fatty infiltration, patient satisfaction, and pain improvement. The time to repair was categorized as 0–2 months, 2–4 months, and more than 4 months. Pain scores improved from 7 to 1.4 (P < 0.01) and active elevation from 55° to 133° (P < 0.01); UCLA and ASES scores improved from 8 to 26 and from 30 to 79, respectively (P < 0.01). All but 2 of the 36 patients were satisfied with the outcome. Preoperative fatty atrophy did not correlate with postoperative function, and tear size did not influence the outcome when repair was performed before 4 months.

Petersen et al. concluded that surgical repair of traumatic rotator cuff tears with associated weakness provided substantial improvement in pain and function, without apparent compromise of results when performed up to 4 months after injury; however, massive tears repaired after this period showed worse outcomes, suggesting greater sensitivity of these lesions to surgical delay.^11^

The general characteristics of the included studies and the certainty of evidence according to the Grading of Recommendations Assessment, Development and Evaluation (GRADE) approach are presented in Tables 1 and 2, respectively.^12,13^

**Table 1:** Characteristics of the included studies.

Included study	Study design	Sample size	Definition of early vs late repair / follow-up
Patel V. et al.^7^	Retrospective cohort	40 patients	Early < 4 months vs late > 4 months; intermediate follow-up
Zhaeentan S. et al.^10^	Prospective cohort	75 shoulders (58 patients)	Early < 3 months (39) vs late > 3 months (36); mean follow-up 56 months
Petersen S.A. et al.^11^	Prospective cohort	36 patients	Timing 0–2, 2–4, and > 4 months; minimum followup 9 months (mean 31)

**Table 2:** Quality of evidence (GRADE): early versus late repair.

Outcome	Studies	Certainty of evidence
Clinical-functional outcomes	3 cohort studies	Very low^1^,^2^,^3^
Re-tear	2 cohort studies	Very low^1^,^2^,^3^
Recovery time / return to pre-injury activities	2 cohort studies	Very low^1^,^2^,^3^

1 Risk of bias (observational design; non-standardized definition of "early" repair);

2 Imprecision;

3 Small sample sizes. A total of 134 patients (151 shoulders) across the three included cohort studies. Note: GRADE (Grading of Recommendations Assessment, Development and Evaluation) is a tool used to assess and grade the certainty of evidence, based on five domains: risk of bias, imprecision, inconsistency, indirectness, and publication bias. The certainty of evidence is classified as very low, low, moderate, or high.

### Study Limitations

Future research should prioritize randomized, multicenter clinical trials with rigorous methodology and a standardized definition of "early" repair. Studies with this design are essential to more precisely define the long-term clinical and functional outcomes associated with each strategy, since no studies with high methodological rigor on this topic were identified.

### Recommendation

Regarding rehabilitation time and the risk of irreparability, there is evidence that early repair (within 3 months of injury) of the rotator cuff is more effective than late repair (after 3 months) for the treatment of traumatic rotator cuff tears.

Therefore, the Brazilian Society of Shoulder and Elbow Surgery (SBCOC) Consensus Project recommends that the surgeon individually assess patient- and disease-related characteristics to reach the best treatment decision. Given the risk of irreparability and longer rehabilitation, this project recommends that, after a traumatic tear, repair be performed as early as possible.

## CONCLUSION

Regarding rehabilitation time and the risk of irreparability, there is evidence that early repair (within 3 months of injury) of the rotator cuff is more effective than late repair (after 3 months) for the treatment of traumatic rotator cuff tears.

## Data Availability

The contents underlying the research are available in the manuscript.

## Appendix 1 Database search strategy.

**Table:**

**PUBMED**
#1 "Rotator Cuff Injuries"[Mesh] OR (Cuff Injury, Rotator) OR (Injury, Rotator Cuff) OR (Rotator Cuff Injury) OR (Rotator Cuff Tears) OR (Rotator Cuff Tear) OR (Tear, Rotator Cuff) OR (Tears, Rotator Cuff) OR (Rotator Cuff Tendinitis) OR (Rotator Cuff Tendinitides) OR (Tendinitis, Rotator Cuff) OR (Rotator Cuff Tendinosis) OR (Rotator Cuff Tendinoses) OR (Tendinoses, Rotator Cuff) OR (Tendinosis, Rotator Cuff) OR (Glenoid Labral Tears) OR (Glenoid Labral Tear) OR (Labral Tear, Glenoid) OR (Labral Tears, Glenoid) OR (Tear, Glenoid Labral)
#2 (Early Rotator Cuff Repair) OR (Early Repair)
#3 (Delayed Rotator Cuff Repair) OR 3 (Delayed Repair)
#4 #1 AND #2 AND #3

**Table:**

**COCHRANE LIBRARY**
#1 (Rotator Cuff Injuries) OR (Cuff Injury, Rotator) OR (Injury, Rotator Cuff) OR (Rotator Cuff Injury) OR (Rotator Cuff Tears) OR (Rotator Cuff Tear) OR (Tear, Rotator Cuff) OR (Tears, Rotator Cuff) OR (Rotator Cuff Tendinitis) OR (Rotator Cuff Tendinitides) OR (Tendinitis, Rotator Cuff) OR (Rotator Cuff Tendinosis) OR (Rotator Cuff Tendinoses) OR (Tendinoses, Rotator Cuff) OR (Tendinosis, Rotator Cuff) OR (Glenoid Labral Tears) OR (Glenoid Labral Tear) OR (Labral Tear, Glenoid) OR (Labral Tears, Glenoid) OR (Tear, Glenoid Labral)
#2 (Early Rotator Cuff Repair) OR (Early Repair)
#3 (Delayed Rotator Cuff Repair) OR 3 (Delayed Repair)
#4 #1 AND #2 AND #3

**Table:**

**EMBASE**
#1 ‘rotator cuff injury’/exp OR ‘cuff injury’ OR ‘cuff lesion’ OR ‘rotator cuff disease’ OR ‘rotator cuff disorder’ OR ‘rotator cuff disorders’ OR ‘rotator cuff injuries’ OR ‘rotator cuff injury’ OR ‘rotator cuff lesion’ OR ‘rotator cuff lesions’ OR ‘rotator cuff pathology’ OR ‘rotator cuff tendinopathies’ OR ‘rotator cuff tendinopathy’ OR ‘shoulder rotator cuff degeneration’ OR ‘shoulder rotator cuff lesion’
#2 (Early Rotator Cuff Repair) OR (Early Repair)
#3 (Delayed Rotator Cuff Repair) OR 3 (Delayed Repair)
#4 #1 AND #2 AND #3

**Table:**

**REGIONAL PORTAL (BVS)**
#1 Mh:"Lesões do Manguito Rotador" OR (Lesões do Manguito Rotador) OR (Lesões do Ligamento Glenoidal) OR (Lesões do Ligamento Glenoídeo) OR (Lesões do Ligamento Glenóideo) OR (Roturas da Coifa dos Rotadores) OR (Roturas do Ligamento Glenoidal) OR (Roturas do Ligamento Glenoídeo) OR (Roturas do Ligamento Glenóideo) OR (Roturas do Manguito Rotador) OR (Ruptura do Manguito Rotador) OR (Rupturas da Coifa dos Rotadores) OR (Rupturas do Ligamento Glenoidal) OR (Rupturas do Ligamento Glenoídeo) OR (Rupturas do Ligamento Glenóideo) OR (Rupturas do Manguito Rotador) OR (Tendinite da Coifa dos Rotadores) OR (Tendinite do Manguito Rotador) OR (Tendinose da Coifa dos Rotadores) OR (Tendinose do Manguito Rotador) OR MH:C26.761.340$ OR MH:C26.803.063$ OR MH:C26.874.400$
#2 (Early Rotator Cuff Repair) OR (Reparo Precoce) OR (Reparación Precoz)
#3 (Delayed Rotator Cuff Repair) OR (Reparo Tardio) OR (Reparación Tardía)
#4 #1 AND #2 AND #3
